# Comparison of complications of early and delayed open reduction and internal fixation for treating pilon fracture: A protocol of systematic review and meta-analysis

**DOI:** 10.1371/journal.pone.0258962

**Published:** 2021-11-18

**Authors:** Yang Chen, Xiaoyu Huang, Yili Chen, Changlong Shi, Hao Li, Jingjing Xu, Yongyao Li, Yachao Du, Yongzhong Cheng

**Affiliations:** 1 Department of Traumatology, Wangjing Hospital of China Academy of Chinese Medical Sciences, Beijing, China; 2 Graduate School of Medicine, Beijing University of Chinese Medicine, Beijing, China; Assiut University Faculty of Medicine, EGYPT

## Abstract

**Background:**

Tibial Pilon fractures are severe fractures accompanied by soft tissue injury. Although open reduction and internal fixation (ORIF) are effective in treating Pilon fractures, there is a controversy over time to surgery due to reported postoperative complications. However, there is no systematic review evaluating the difference of postoperative complications between early and delayed ORIF for treating pilon fractures.

**Methods:**

Relevant literature written in English will be searched through PubMed, Cochrane Library, Embase, MEDLINE, and Web of Science. The study aims to compare the effects and complications of early and delayed ORIF for treating fresh pilon fractures in adult patients. The primary outcome will be infection rate, fracture union time, nonunion and malunion rate. And the secondary outcome will be metalwork removal, amputation, and ankle function grade. Two reviewers will independently assess the eligibility of the studies according to the pre-defined inclusion and exclusion criteria. A meta-analysis for the available data will be conducted using Revman 5.3. To measure effect size, odds ratios (ORs) and mean difference will be used for dichotomous and continuous data, respectively. Statistical heterogeneity will be explored. And a random-effects model or a fixed-effects will be used in pooled data on the basis of the existence or absence of heterogeneity. Subgroup analysis will be conducted to identify sources of heterogeneity and sensitivity analysis to test the results’ robustness. We will assess the risk of bias by four different quality assessment tools according to the study design. Publication bias will be evaluated by funnel plot. The study data will be stored in the Open Science Framework website.

**PROSPERO registration number:**

CRD42020207465

## 1. Introduction

Tibial Pilon fractures, defined as fractures of distal tibial extending to the joint surface, account for 1% of all lower limb fractures [1]. They are commonly caused by high-energy axial forces and rotational forces. The combinations of articular comminution and metadiaphyseal osseous deficits result in difficulty of fracture reduction, which is a challenge for surgeons [2–4]. Additionally, because of extensive soft tissue damage in high-energy trauma, there are high risks of wound infection, nonunion, and delayed union. Posttraumatic arthritis is also a common complication of pilon fractures [5, 6].

Management of pilon fracture aims not only to achieve anatomical reduction with the restoration of mechanical axis and length of the lower limbs and joint congruity, fixation stable enough for exercises, and early functional mobility but also to prevent complications [7]. The complication rate, however, is relevant to soft tissue conditions, comorbidities, time to operation, and surgical intervention approach [8–10]. Early ORIF, known as a one-stage procedure with definitive internal fixation, which was reported by Ruedi and Allgower in 1969 with good outcomes in pilon fracture [11]. Although the anatomical reduction is achieved, other orthopedic surgeons are faced with a higher complication rate. Literature reported that infection rate ranges from 0% to 55% [12]. However, recently a cohort study documented that the infection rate of early ORIF is only 2.7% and a good reduction rate is 90% [13]. Two-stage open reduction and internal fixation, proposed by Patterson and Cole, are regarded as a method that protects soft tissue and reduces infection rate [14]. This approach involves temporary fixation with external fixators or calcaneal traction, followed by open reduction and internal fixation. However, recent studies have suggested that the time of surgery has few correlations with wound complications, and delayed surgery may increase risk of infection [11, 15]. Moreover, this method leads to a long-term hospital stay and lack of anatomical reduction due to delayed operation [16].

## 2. Objective

The aim of the systematic review is to answer the PICO question: “Do early and delayed open reduction and internal fixation have different influence on postoperative complications in adult patients with pilon fracture?” The relationship between time to surgical and complication will be discussed to conclude the differences in complications between the two treatment options.

## 3. Methods

The protocol of this systematic review and meta-analysis has been registered in PROSPERO and will be conducted in line with the Preferred Reporting Items for Systematic Reviews and Meta-Analyses (PRISMA) guidelines [17]. ethical approvals and patient consent are not necessary because the meta-analysis will be based on published research. We will submit our meta-analysis to a peer-reviewed journal for publication.

### 3.1 Eligibility criteria

#### 3.1.1 Types of studies

All randomized controlled trials (RCTs) and non-randomized studies (cohort studies or case-series) will be included. The included studies will be written in English. Reviews, case reports, conference abstracts, letters, the study protocol will also be excluded.

#### 3.1.2 Types of participants

Participants will be those diagnosed with pilon fractures by radiology. Only adult patients older than 18 will be included. Studies investigating patients with old pilon fractures will be excluded. Old fracture is defined as fracture with the interval between consulting time and injury time exceeding three weeks. There was no restriction on gender and race.

#### 3.1.3 Types of interventions

The surgical approach will be early or delayed ORIF. Early ORIF refers to the one-stage procedure with definitive internal fixation. Delayed ORIF is a temporary fixation of external fixation followed by open reduction and internal fixation.

#### 3.1.4 Outcomes

Primary outcomes: superficial infection and deep infection rate; fracture union time.

Secondary outcomes: nonunion (defined as fracture with a healing time of more than 9 months), metalwork removal, amputation, ankle function grade.

### 3.2 Search strategy

PubMed, Cochrane Library, Embase, MEDLINE, Web of Science will be searched for literature written in English. Time will be set from the inception of the database to the day on which the search is performed.

We plan to search for literature by free word. Before systemic review and meta-analysis, the database will be searched on a regular basis, and references will be screened to identify additional sources. Final search will be performed before full-text were finished to update results. An example search strategy for Cochrane Library is shown in Table 1.

**Table 1:** Search strategy for Cochrane Library.

Search term	
1#	Pilon OR tibial plafond fracture OR distal tibial fracture OR tibial pilon fracture
2#	Primary fixation OR early fixation OR one-stage
3#	delayed fixation OR two-stage treatment
4#	1# AND 2# AND 3#

### 3.3 Study selection, data extraction, and management

**Fig 1:**
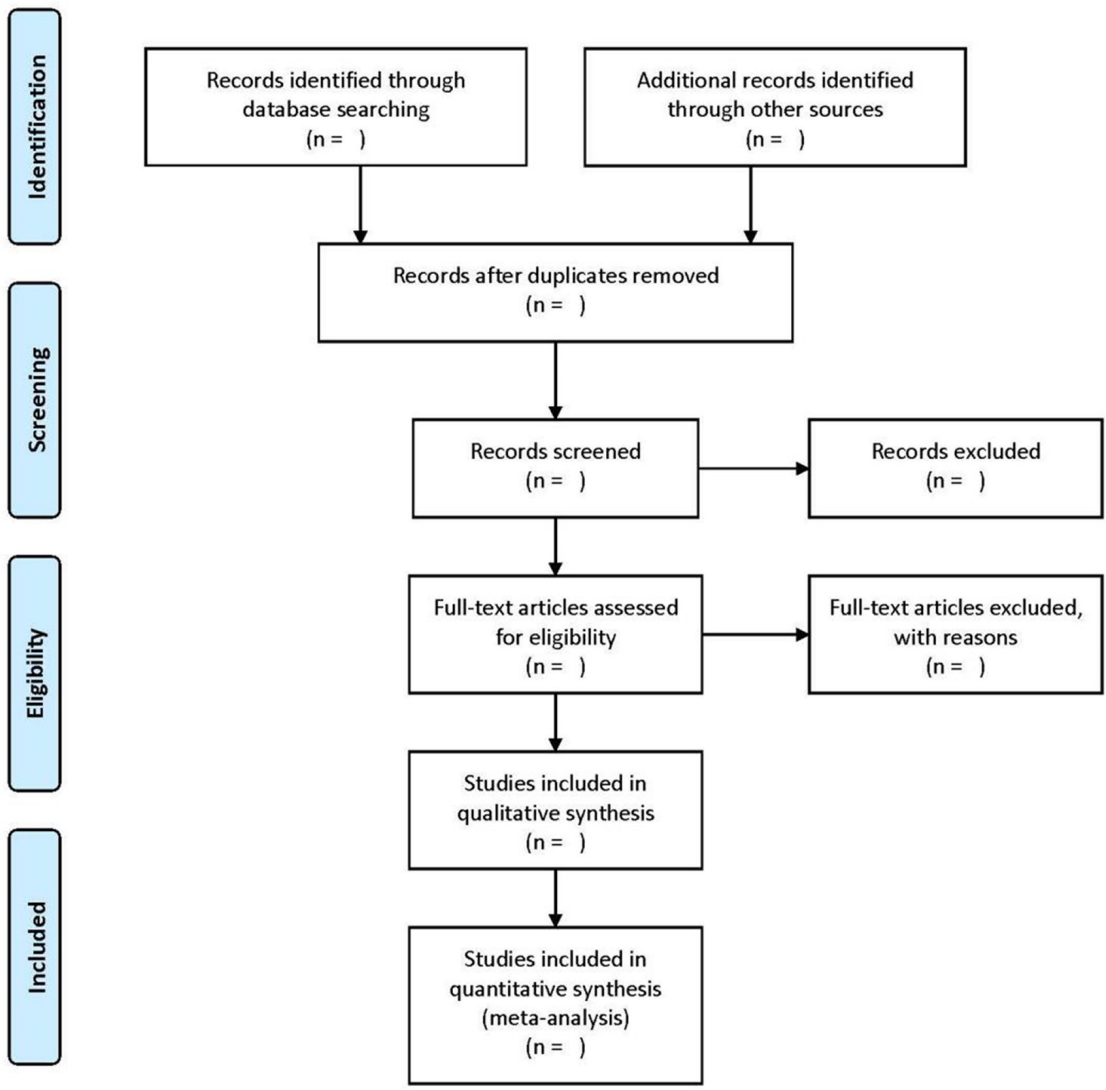
A flow chart for the systematic review.

Two reviewers will independently assess the eligibility of the studies according to the pre-defined inclusion and exclusion criteria. After duplications from all the search citations being removed, titles and abstracts of the remaining ones will be screened for their eligibility. The full texts will then be retrieved to determine further if they meet the inclusion and exclusion criteria. Any disagreement will be resolved by the third author. The process of identifying, screening, and including and excluding results will be shown using the flow chart (Fig 1).

Data will be independently extracted by two authors using a pre-designed standard form involving patient age and gender, fracture type and pilon fracture classification, intervention and operative description, complications encountered, radiological and clinical time to union, ankle function, and documented length of follow-up. Missing information will be obtained by consulting the corresponding author of original studies.

We will upload our protocols, data extraction information, and analytic results on the Open Science Framework website for researchers to control/prove our findings. (https://help.osf.io/hc/en-us).

### 3.4 Assessment of risk of bias

Two review authors will independently evaluate the risk of bias of included studies. The risk of bias of randomized controlled trials (RCTs) will be rated using the Cochrane quality assessment tool for RCTs. Risk of bias in non-randomized studies of Intervention (ROBINS-I) tool will be used to assess the risk of bias in the included non-randomized studies, and the Newcastle–Ottawa scale (NOS) [18] is planned to be used to assess the quality of case-control and cohort studies. A third reviewer will be introduced as an arbitrator if there is any disagreement among the two review authors. Case-series will be assessed by quality appraisal checklist of case series studies, which was put forward by Institute of Health Economics [19].

### 3.5 Statistical analysis

If data attracted from eligible literature is available for meta-analyses, Review Manager 5.3 software will be used. Statistical heterogeneity will be determined by I² statistic [20] with significance set at ≥50%, and the Chi^2^ statistic [21] with significance set at P < 0.10. When significant heterogeneity exists, a random-effects model will be used, otherwise, a fixed-effects model will be utilized.

If RCTs or non-randomized studies are not included, or if the heterogeneity among studies making meta-analysis impossible, the descriptive analysis will be performed. We plan to assess possible sources of heterogeneity by subgroup and sensitivity analyses where data are available.

In the condition of missing or unclear data, study authors will be contacted at the data extraction stage. If data are not acquired, we consider that existing data will be analyzed. The relevant factor will be used to calculate the continuous variable of standard deviation.

### 3.6 Subgroup analysis

Subgroup analysis will be performed if we want to find the source of heterogeneity among these studies. Fracture classification, compromise of soft tissue, and literature quality will be considered in subgroups of analysis.

### 3.7 Sensitivity analysis

A sensitivity analysis will be performed to confirm whether the pooled results are robust and credible by excluding highly biased studies or including articles one by one.

### 3.8 Publication bias

Publication bias will be evaluated by funnel plot (RevMan). If the number of studies exceeds 10, Egger’s test (Stata) will be used to estimate bias quantification.

### 3.9 Evidence evaluation

The Grading of Recommendations Assessment, Development, and Evaluation system (GRADE) [22] will be used to evaluate the strength of evidence for each outcome.

## 4. Discussion

The currently available literature has not reached a consensus regarding which of early ORIF and delay ORIF result in less postoperative complications. Therefore, we plan to perform the systemic review and meta-analysis to present a general view of the current literature and quantitatively analyze the data of studies.

To our knowledge, this will be the first systemic review and meta-analysis investigating the influence of early ORIF versus delay ORIF on postoperative complications. We expect to demonstrate results of the study in several ways. Firstly, we will show infection rate of two surgical ways, including superficial infection and deep infection. Infection is an important factor for assessment of therapeutic effect and can result in skin necrosis and exposure of internal plant [23]. Secondly, time to union, delayed union and nonunion rate will be documented, which are relevant to time of weight-bearing exercise and return to work and activities. Finally, because metalwork removal rate and ankle function grade reflect re-operation rate and quality of life [24], we will present the results in our study. In a word, the study’s findings could provide orthopedists with evidence of the optimal operation time of treatment for pilon fracture. Moreover, it may also demonstrate the hypothesis of the window period of soft tissue.

There are some limitations in this study. Firstly, although we plan to search multidisciplinary databases, some specific databases are likely to be omitted, which can produce bias. Additionally, when data of randomized controlled trials (RCTs) and non-randomized studies are pooled, heterogeneity of articles can have negative effect on conclusion. The Cochrane Handbook for Systematic Reviews of Interventions recommendations will be used and data of literatures of different categories will be pooled respectively to minimize these limitations.

### 4.1 Amendments

If amendments are necessary, we will state the alteration and reasons, along with each amendment date.

## Supporting information

S1 Checklist (DOC)

## Abbreviations

ORIF: open reduction and internal fixation
